# Evolution of ROP screening at Aravind Eye Hospital, Coimbatore - Lessons learnt and the way ahead

**Published:** 2017-11-11

**Authors:** Parag K. Shah, V. Narendran, N. Kalpana

**Affiliations:** 1 Consultant: Department of Pediatric Retina & Ocular Oncology, Aravind Eye Hospital & Post Graduate Institute of Ophthalmology, Coimbatore, India.; 2 Chief Medical Officer: Department of Pediatric Retina & Ocular Oncology, Aravind Eye Hospital & Post Graduate Institute of Ophthalmology, Coimbatore, India.; 3 Senior Medical Officer: Department of Pediatric Retina & Ocular Oncology, Aravind Eye Hospital & Post Graduate Institute of Ophthalmology, Coimbatore, India.

**Through Aravind ROP Tele-screening Project called Retinopathy of Prematurity Eradication Save Our Sight (ROPE-SOS), 8117 babies were screened and 127 babies were treated between 2015 and 2017.**

## ROP in India

According to World Health Organization (WHO) report, there are 15 million preterm births (<37 weeks) per year in the world, and annually India has the largest number of premature babies (3,59,100).^1^ With improving economies, the neonatal care facilities are also improving and consequently survival rate of premature babies has increased. With a birth rate of 23 per 1000 population and about 12% of infants being born prematurely in India, it is estimated that incidence of ROP is 20–30%.^2^ India also has a highest risk of ROP blindness due to sub-optimal neonatal care and lack of screening facilities.^3^ ROP is rapidly becoming a public health issue as the screening and treatment services are estimated to be 30% lower than the present need.^4^

## ROP services at Aravind Eye Hospital, Coimbatore

ROP screening was started at Aravind Eye Hospital, Coimbatore in the year 2000 by the paediatric ophthalmology department. Initially screening started covering a single neonatal intensive care unit (NICU) once a week. Babies who needed laser treatment were treated with green laser (532 nm). From 2002 the retina department took over the ROP screening services and since then, on a weekly basis a retina specialist visited the NICU. From 2003 more NICUs were added and currently Aravind Hospital covers eight major NICUs in Coimbatore.

**Figure 1:**
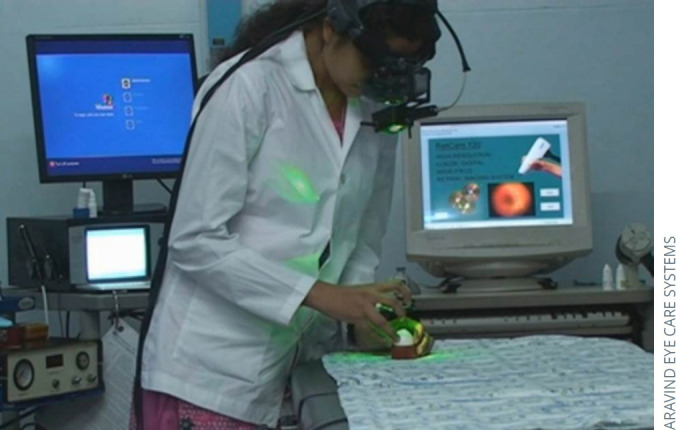
ROP trainee practising indirect laser on RetiEye.

Aravind Coimbatore was the first institute in India to get the RetCam 120 digital fundus imaging camera in 2003 and infra-red diode (810 nm) laser was also added in the following year. Use of intra-vitreal injection of anti-vascular endothelial growth factor (VEGF) was introduced in 2006. As the number of ROP cases increased over a period of time, a separate Paediatric Retina Clinic was inaugurated. A month-long ROP training programme was initiated, wherein candidates are trained to examine infants using indirect ophalmoscopy and practice indirect laser on the RetiEye Model eye (Aurolab, Madurai, India) (Figure 1). So far 54 candidates from India and abroad have been trained under this programme, 41 from India, five from African countries and eight from a variety of other countries.

**Figure 2:**
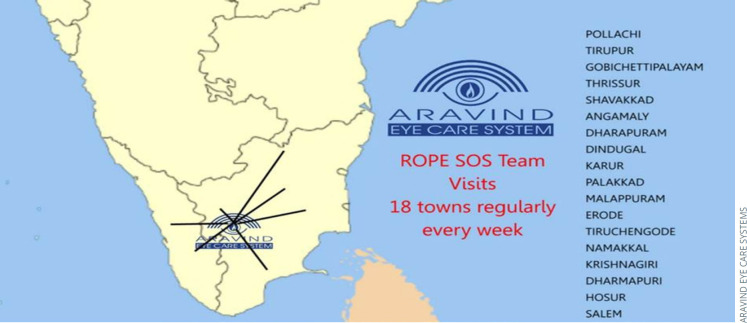
Cities covered in the states of Tamil Nadu and Kerala under the ROPE-SOS Tele-screening project.

To serve the unreached in rural areas, Aravind ROP Tele-screening Project called Retinopathy of Prematurity Eradication Save Our Sight (ROPE-SOS) was launched in August 2015. The project aimed to screen 2000 babies per year in the sub-urban and rural areas. Technicians are trained to capture fundus images of pre-term babies with help of digital retinal camera (RetCam). The team comprises of one manager, two trained technicians, one mid-level ophthalmic assistant and a driver. The team covers 56 NICUs of 18 cities in 12 districts of Tamil Nadu and Kerala (Figure 2). The team visits scheduled district hospital NICU on specified days in a customised van with a RetCam shuttle. The technicians enter the details of the babies in RetCam and obtain fundus images. The digital images of the fundus are then transmitted to the base hospital through broadband internet.

The indigenously developed Aravind Diabetic Retinopathy Eye Screening (ADRES) software is used to transmit these images. The ADRES software was modified for ROP. At the base hospital, images are graded by a ROP expert (retinal specialist) and the report is sent back immediately to the NICU. The 4G network (which is now available in remote parts of India) is used to transfer these images. The family is explained about the baby's eye status and given a follow-up date. The whole process for screening and counselling parents takes about 12–15 minutes per baby. If a baby requires treatment and if the baby is stable systemically, the baby is transferred to Aravind Eye Hospital Coimbatore for management. If the baby is not stable for distant travel, the ROP expert visits the NICU within three days to provide treatment. With the help of tele-screening, various other eye conditions like cataract, corneal opacity and even retinoblastoma have been diagnosed and promptly managed by early referral.

Through this mode of screening from August 2015 to June 2017, 8117 babies were screened and 127 babies were treated. By including anterior segment photography 10 babies underwent cataract surgery which was diagnosed by tele-screening.

**Figure 3:**
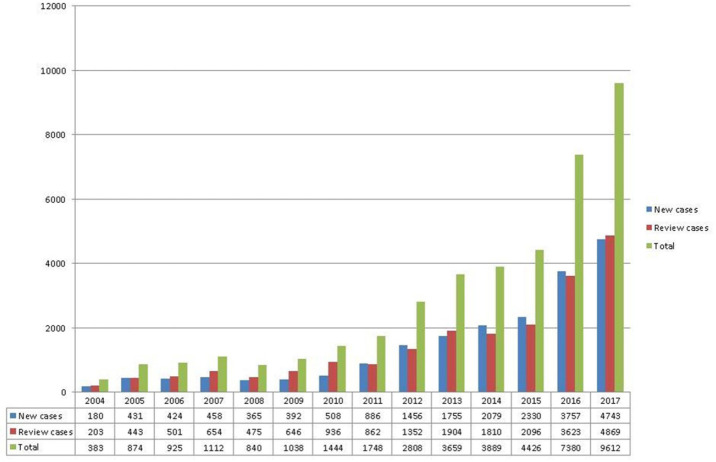
Bar diagram showing a steady increase in ROP cases in Aravind Eye Hospital, Coimbatore over a period of 13 years (2004 – 2017).

With this process about 127 babies were prevented from going blind due to ROP in the last two years. The total growth of ROP screening from 2003 to 2017 is shown in Figure 3. As part of the ROPE-SOS project, awareness of ROP and the importance of screening is spread by means of continued medical education (CME) programmes conducted in the districts screened. The CME programme spreads awareness of ROP, the impact of external factors like oxygen on disease, the ideal time for screening and indication for screening. The neonatal nurses and neonatologist are targeted in all CME programmes. So far CME programmes were held in 11 cities sensitising 711 NICU staff. Of these 233 were nurses and 31 were paediatricians and neonatologists. Patient information posters and brochures were displayed and distributed widely. With the success of ROPE SOS project, it is now being replicated at Aravind Eye Hospital, Tirunelveli. Vitrectomies for advanced ROP are done using the 25 or 27 gauge instruments. With lack of surgical training in ROP, a one year long term Surgical Paediatric Retina Fellowship was also launched in 2016.

## Conclusion

The journey of starting ROP services at Aravind Eye Hospital, Coimbatore has been quite satisfactory and with the tools for screening and management in place, mentoring other upcoming institutes in India and abroad is on-going. Developing automated diagnosis of ROP using computer assisted deep learning is the next goal.^5^
